# Integrating Human Proteomes with Genome-Wide Association Data Reveals Prioritized Therapeutic Candidates for Lung Squamous Cell Carcinoma

**DOI:** 10.3390/biology14121640

**Published:** 2025-11-21

**Authors:** Yutong Zhang, Yiran Zhao, Lingli Fan, Xiaoyan Li, Yuanyuan Li

**Affiliations:** 1 School of Life Sciences and Medical Engineering, Anhui University, Hefei 230601, Chinalixiaoyan@ahu.edu.cn (X.L.); 2 Institutes of Physical Science and Information Technology, Anhui University, Hefei 230601, China

**Keywords:** lung squamous cell carcinoma, drug target, Mendelian randomization, pQTL, GWAS

## Abstract

Lung squamous cell carcinoma (LUSC) is the second most prevalent type of lung cancer worldwide. Despite its global health burden, the molecular mechanisms driving LUSC remain poorly characterized, posing considerable challenges for the development of targeted preventive therapies. Here, by integrating human plasma proteomes (*n* = 54,219) with GWAS summary data for LUSC (7426 cases and 55,627 controls), we performed genome-wide Mendelian randomization (MR) and colocalization analyses to identify potential druggable targets associated with LUSC risk. After applying Bonferroni correction, sensitivity analyses, and reverse causation detection, we identified 12 potential druggable proteins significantly associated with LUSC risk. Five of these proteins (DOK2, FKBPL, NCF2, PDIA3, and TCL1A) showed strong evidence of colocalization. Furthermore, protein–protein interaction (PPI) networks and druggability assessments were used to refine therapeutic target selection. Additionally, mediation analyses were performed to elucidate the mediating effects of modifiable risk factors on the relationship between plasma proteins and LUSC risk, and we identified 14 modifiable risk factors that could mitigate LUSC risk through targeted interventions. More importantly, we stratified the 12 proteins into four tiers based on colocalization, differential expression, PPI networks, and druggability potential. Notably, DOK2 emerged as a Tier 1 target, while FKBPL, NCF2, AXL, and PDIA3 were classified as Tier 2 targets, representing promising candidates for further drug development. Overall, we identified 12 proteins with druggable potential associated with LUSC risk and demonstrated how modifiable risk factors mediate these associations. These findings advance our understanding of LUSC etiology and provide a foundation for developing targeted therapeutic strategies while emphasizing the importance of addressing modifiable risk factors in both prevention and treatment efforts.

## Simple Summary

Lung squamous cell carcinoma (LUSC) represents a clinically challenging subtype of non-small-cell lung cancer, where targeted treatment options remain limited. To address this unmet need, we systematically investigated the causal roles of blood plasma proteins in LUSC development by integrating genetic data from large-scale studies. Using a Mendelian randomization (MR) approach that leverages natural genetic variation as instrumental variables, we identified twelve circulating proteins whose levels are causally linked to LUSC risk. Among these, five proteins (including DOK2, FKBPL, NCF2, PDIA3, and TCL1A) exhibited strong evidence of shared genetic architecture with LUSC. We further classified these candidates into prioritized tiers based on colocalization, differential gene expression, interaction networks, and druggability profiles. In addition, mediation analysis revealed that modifiable risk factors, such as smoking and physical activity, partly influence the protein–LUSC relationship. Our study provides a genetically validated roadmap for targeting novel therapeutic candidates and supports the integration of lifestyle interventions in LUSC prevention and treatment strategies.

## 1. Introduction

Lung squamous cell carcinoma (LUSC), a major subtype of non-small-cell lung cancer (NSCLC), accounts for approximately 25–30% of NSCLC cases and remains a leading cause of cancer-related mortality worldwide [1,2]. Although significant progress has been made in the treatment of lung adenocarcinoma, another subtype of NSCLC, through molecularly targeted therapies, such as those targeting the epidermal growth factor receptor (EGFR) and anaplastic lymphoma kinase (ALK) [3,4,5,6], similar advancements have not been achieved for LUSC. Currently, treatment options for LUSC predominantly rely on traditional therapeutic modalities, including surgery, chemotherapy, and radiotherapy [7]. The lack of approved molecularly targeted therapies underscores the urgent need to identify potential therapeutic drug targets for LUSC.

Proteins, as critical regulators of a range of complex biological processes, represent a cornerstone of human cellular function and an important therapeutic target for the development of anticancer drugs [8]. Advances in high-throughput proteomics have facilitated the identification of aberrant proteins associated with various malignancies, offering valuable insights into drug target discovery. Although genome-wide association studies (GWAS) have uncovered multiple genetic loci associated with LUSC [9,10], the causal genes within these loci and their molecular mechanisms remain poorly understood, limiting their translational potential. Thus, additional analyses are needed to clarify the causal genes within these susceptibility regions and elucidate their roles in LUSC pathogenesis.

Accumulating evidence has highlighted the importance of genetic support for protein drug targets in enhancing the success rate of drug development [11]. Mendelian randomization (MR), an emerging and powerful approach for causal inference, has achieved great success in drug target development and drug repurposing [12]. By leveraging genetic variants, such as single nucleotide polymorphisms (SNPs), as instrumental variables (IVs), MR can assess the causal relationship between exposure factors and outcomes, mitigating biases from confounding factors and reverse causality [13]. Integrating protein quantitative trait loci (pQTLs) with GWAS summary data through MR provides an effective strategy for identifying druggable proteins in LUSC.

In this study, we conducted a comprehensive proteome-wide MR analysis, integrating human plasma proteomic data to identify potential drug targets for LUSC. A total of 12 proteins with druggable potential were identified as significantly associated with LUSC risk. Bayesian colocalization analysis provided strong genetic evidence supporting five of these proteins as promising therapeutic candidates. To validate these findings, we performed differential expression analysis to assess the presence of candidate proteins in LUSC tissues, establishing their biological relevance. Protein–protein interaction (PPI) networks were employed to evaluate functional relationships between the identified proteins and known LUSC drug targets, uncovering key interactions. Additionally, mediation analyses evaluated the causal effects of pathogenic plasma proteins on modifiable risk factors associated with LUSC and clarified the extent to which these factors mediated the influence of candidate proteins on LUSC risk. Overall, our approach prioritized 12 high-confidence susceptibility proteins linked to LUSC risk, offering a valuable resource for the development of targeted therapies and providing new insights into precision medicine strategies for this disease.

## 2. Materials and Methods

### 2.1. GWAS and Plasma pQTL Data

The largest GWAS summary statistics for LUSC [14] were obtained from the Transdisciplinary Research Consortium for Lung Cancer (TRICL), encompassing data from 7426 LUSC cases and 55,627 controls. These statistics, originally reported by McKay et al., were derived from a fixed effects meta-analysis of GWAS data involving 29,266 clinically diagnosed lung cancer cases and 56,450 controls [14,15,16,17]. The study identified 18 susceptibility loci, with additional analyses stratified by histological subtype (small cell carcinoma, LUSC, and adenocarcinoma) where data were available [14]. For the current study, only GWAS summary statistics specific to LUSC were included. Details regarding sample collection, quality control, and data analysis are available in the original publication [14].

The plasma pQTL data were sourced from the UK Biobank Pharma Proteomics Project (UKB-PPP), which involved 54,219 participants [18]. This comprehensive dataset mapped 2923 proteins, identifying 14,287 primary genetic associations, 81% of which were novel. To ensure robust data selection, the following criteria were applied: (i) genome-wide significance reaching *p* < 5 × 10^−8^; (ii) exclusion of the major histocompatibility complex (MHC) region (chr6, 26–34 Mb on hg19); (iii) selection of independent associations using linkage disequilibrium (LD) clumping (r^2^ < 0.001); and (iv) only *cis*-acting pQTLs. This process yielded 6202 SNPs significantly associated with 1972 proteins (Supplementary Table S1).

### 2.2. MR Analysis

To investigate the association between genetically predicted protein expression and LUSC risk, primary MR analyses were performed using the R package “TwoSampleMR” (v0.6.1) [19,20]. Plasma pQTL data served as the exposure, while LUSC GWAS summary statistics represented the outcome. For proteins with a single available pQTL (i.e., IV = 1), the Wald ratio method was applied to estimate the relationship between genetically proxied protein expression and LUSC risk. When multiple genetic IVs were available for a given protein, advanced MR methods, including inverse variance weighting (IVW), MR-Egger regression, weighted median, weighted mode, and simple mode approaches, were used to ensure comprehensive and accurate causal inference. Among these, IVW is regarded as the most statistically robust approach for deriving MR estimates using multiple IVs [19,20,21].

To account for multiple testing, Bonferroni correction was employed, setting a stringent threshold of *p* < 2.54 × 10^−5^ (0.05/1972) for identifying potential causal proteins. Heterogeneity was evaluated using Cochran’s Q test [22], while pleiotropy was assessed using the MR-Egger regression intercept [23]. An odds ratio (OR) exceeding 1 indicated that increased levels of plasma protein expression may increase LUSC risk. This analytical framework allowed for the robust identification of genetically proxied plasma proteins with potential causal roles in LUSC development. To visually assess the robustness and potential pleiotropy of the MR estimates, we generated scatter plots, leave-one-out sensitivity analysis plots, and funnel plots for all significant associations.

### 2.3. Reverse Causality Detection

In complex biological systems, reverse causality can lead to incorrect causal inferences when an IV affects exposure via the outcome. To ensure robust causal inferences, bidirectional MR analysis were performed using LUSC as the exposure and pQTL as the outcome. The screening and analytical procedures adhered to the methodology established in the primary MR analyses. This bidirectional approach facilitated the detection of potential reverse causality, thereby reducing the likelihood of confounded results. Statistical significance was determined at a threshold of *p* < 0.05.

To further address reverse causality, Steiger filtering was applied to exclude IVs that demonstrated stronger associations with the outcome than with the exposure [24]. Plasma proteins showing evidence of reverse causality were excluded from further analyses. Statistical significance was determined at a threshold of *p* < 0.05.

### 2.4. Bayesian Colocalization Analysis

Bayesian colocalization analysis was performed to evaluate whether the genetic variants associated with protein expression and LUSC risk were shared, thus distinguishing true associations from confounding due to LD [25]. Analysis was conducted using the R package “coloc” (v5.3.2) with default parameters to estimate the posterior probability of a shared causal variant between cis-pQTLs (± 500 kb) and LUSC GWAS signals.

The Bayesian colocalization framework provided posterior probabilities for five hypotheses [25,26]: H0, no association with either plasma protein or LUAD; H1, association with plasma proteins only; H2, association with LUSC only; H3, association with both plasma proteins and LUSC, driven by distinct causal variants; H4, association with both plasma proteins and LUSC, driven by a shared genetic variant. Regions with a posterior probability for H4 (PPH4) ≥ 0.75 were considered to provide strong evidence for a shared causal genetic variant, while PPH4 ≥ 0.60 indicated moderate support for a causal protein.

### 2.5. Protein–Protein Interaction Network (PPI)

To investigate the interactions between the identified plasma proteins and established therapeutic targets for LUSC, previously recognized LUSC drugs were obtained from the literature [27,28,29], and their corresponding target information was extracted from the DrugBank database (https://go.drugbank.com/; accessed on 1 June 2024) [29]. A PPI analysis was then conducted to evaluate the relationships between the identified plasma proteins and current therapeutic targets [30], using the STRING database (https://string-db.org; accessed on 1 June 2024). A minimum required interaction score of 0.40 was applied to ensure reliable interaction predictions.

### 2.6. Evaluation of Druggability

The druggability of the identified target proteins was assessed to prioritize potential therapeutic candidates for LUSC. Druggability evaluation was performed using data from the ChEMBL (https://www.ebi.ac.uk/chembl; accessed on 1 June 2024) [30], DGIdb (https://dgidb.org/; accessed on 1 June 2024) [31] and DrugBank databases [32]. These databases were selected for their comprehensive coverage of drug-gene interactions, functional annotations, and expert-reviewed evidence.

### 2.7. Differential Expression Analysis

To determine whether the identified protein-coding genes were dysregulated in LUSC patients, gene expression profiles and corresponding clinical data were retrieved from the Gene Expression Omnibus (GEO) database (accession number GSE30219) [33] (https://www.ncbi.nlm.nih.gov/geo/; accessed on 1 June 2024) and the Cancer Genome Atlas (TCGA) database (https://portal.gdc.cancer.gov; accessed on 1 June 2024). The GSE30219 dataset included gene expression data from 61 LUSC samples and 14 normal tissue samples, while the TCGA-LUSC dataset provided bulk RNA-seq data from 502 LUSC samples and 51 LUSC adjacent normal tissue samples.

Differential expression analysis was performed using the limma (Linear Models for Microarray Data) package (v3.58.1) [34]. Genes were considered significantly differentially expressed if they met the criteria: |Log2 fold change (Log2FC)| > 1 and adjusted *p* < 0.05.

### 2.8. Mediation Analysis

Candidate risk factors for LUSC were identified through an extensive literature review [19,35]. Smoking status emerged as a primary contributor to the development and progression of LUSC. Additional risk factors potentially influencing LUSC developed included diet, lifestyle behaviors, physical activity, alcohol consumption, genetic susceptibility, and chronic obstructive pulmonary disease (COPD). Risk factors without available GWAS data were excluded, resulting in the inclusion of 34 modifiable risk factors. Details on the GWAS summary data for these modifiable risk factors are provided in Supplementary Table S2.

To identify the mechanisms linking identified proteins to LUSC risk, mediation analyses were conducted using a two-step MR approach [36]. First, two-sample MR analyses were performed to assess causal associations between plasma proteins and modifiable risk factors. Subsequently, the causal effects of these risk factors on LUSC were evaluated, providing a comprehensive understanding of the role of proteins in LUSC development [37]. Mediating effects were quantified using the Product method, with the Delta method employed to determine the standard error (SE) and confidence interval (CI) [38]. Risk factors were considered mediators if they met two criteria: (i) significance (*p* < 0.05) in both MR steps, and (ii) consistency in the direction of the mediating and total effects, as determined by the primary MR analysis. A nominal significance threshold was applied to optimize the identification of potential modifiable mediators. This approach elucidated pathways through which plasma proteins influence LUSC risk, highlighting actionable targets for prevention and intervention.

### 2.9. Classification of Proteins into Evidence-Based Tiers

Based on the integrated evidence from colocalization, differential expression, PPI, and druggability assessment, the 12 candidate proteins were classified into four tiers. Proteins in Tier 1 were required to meet all of the following criteria: strong colocalization evidence (PPH4 > 0.75), significant differential expression in LUSC tissues, presence in the PPI network with known LUSC drug targets, and documented druggability potential. Tier 2 proteins satisfied at least three of these criteria. Tier 3 proteins met at least two criteria, while Tier 4 included proteins with only a significant MR association (*p* < 2.54 × 10^−5^) but lacking strong support from the other evidence types. This multi-dimensional stratification allowed for a systematic prioritization of targets based on the consistency and strength of genetic, functional, and therapeutic evidence.

## 3. Results

### 3.1. Integrative Analysis of pQTL Data Prioritized 12 Plasma Proteins Associated with LUSC Risk

**Figure 1:**
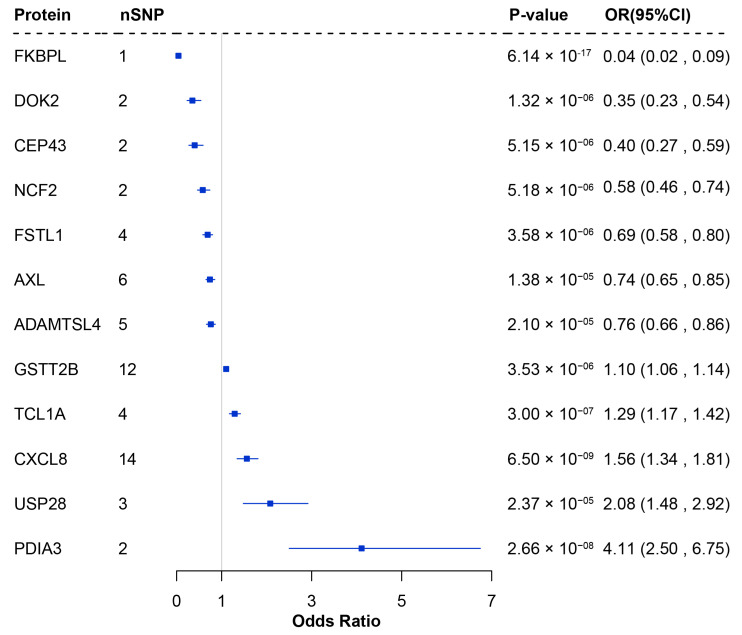
Forest plot of proteins significantly associated with LUSC in the proteome-wide MR analysis. nSNP: Number of SNP used for the estimation of the causal effects; CI: Confidence interval; OR: Odds ratio; MR: Mendelian randomization.

By integrating pQTL data from 54,219 UK Biobank participants with LUSC GWAS summary statistics from the TRICL consortium, we conducted a primary MR analysis to explore the causal relationships between 1972 plasma proteins and LUSC risk. Following Bonferroni correction (*p* < 2.54 × 10^−5^), 12 plasma proteins were identified as significantly associated with LUSC, including ADAMTS-like protein 4 (ADAMTSL4), tyrosine-protein kinase receptor UFO (AXL), centrosomal protein 43 (CEP43), interleukin-8 (CXCL8), docking protein 2 (DOK2), FK506-binding protein-like (FKBPL), follistatin-related protein 1 (FSTL1), glutathione S-transferase theta-2B (GSTT2B), neutrophil cytosol factor 2 (NCF2), protein disulfide-isomerase A3 (PDIA3), T-cell leukemia/lymphoma protein 1A (TCL1A), and ubiquitin carboxyl-terminal hydrolase 28 (USP28). Among these, reduced expression of seven proteins (ADAMTSL4, AXL, CEP43, DOK2, FKBPL, FSTL1, and NCF2) was associated with increased LUSC risk, while elevated expression of five proteins (CXCL8, GSTT2B, TCL1A, PDIA3, and USP28) was linked to increased LUSC risk (Figure 1 and Supplementary Table S3).

### 3.2. Quality Control Identified Five Candidate Protein Targets with Robust MR Evidence

The robustness of the primary MR analysis was confirmed through comprehensive sensitivity analyses. No evidence of heterogeneity or pleiotropy effects was observed, as all 12 proteins met the criteria P_Q-stat_ > 0.05 and P_Egger-Intercept_ > 0.05 (Supplementary Table S4). Bidirectional MR analyses provided no indication of reverse causality, demonstrating that LUSC did not influence protein expression (Supplementary Table S5). Additionally, Steiger filtering confirmed the causal directions, reinforcing the validity of the observed associations. These findings suggest that the identified proteins maintain robust causal links to LUSC, unaffected by confounding risk factors. Furthermore, visual inspection of scatter, leave-one-out, and funnel plots (Supplementary Figures S1–S6) confirmed the robustness of these causal associations, showing no evidence of influential outliers or substantial directional pleiotropy.

To further evaluate the genetic basis of the associations, colocalization analysis was performed to determine whether a shared genetic signal (i.e., IV) drives the association between protein expression and LUSC. Strong evidence (PPH4 > 0.75) supported colocalization for five proteins—DOK2, FKBPL, NCF2, PDIA3, and TCL1A—indicating shared causal variants associated with LUSC (Supplementary Table S6). Moderate colocalization support (0.50 < PPH4 < 0.75) was observed for CEP43. Notably, proteins with strong colocalization evidence are more likely to serve as viable drug targets, emphasizing their therapeutic potential [39].

### 3.3. Differential Expression Analysis of MR-Identified Protein-Coding Genes

To further determine whether the protein-coding genes identified by MR analysis were dysregulated in LUSC patients compared to healthy tissues, differential expression analysis was conducted using gene expression profiles from the GEO and TCGA datasets. Among the 12 protein-coding genes, six were significantly dysregulated in LUSC tissues in either the GEO or TCGA datasets (Supplementary Figure S7). Notably, four genes (*ADAMTSL4*, *AXL*, *DOK2*, and *NCF2*) showed consistently dysregulation across both datasets, with concordant effect directions (Supplementary Tables S7 and S8), strongly supporting their potential as therapeutic targets for LUSC. The remaining two genes (*FKBPL* and *USP28*) were differentially expressed only in the TCGA dataset. Furthermore, we have compared the direction of effects between the MR and differential expression analyses for the four overlapping genes (*ADAMTSL4*, *AXL*, *DOK2*, and *NCF2*). Specifically, the MR analysis showed that the decreased protein expression level is associated with an increased risk of LUSC (OR < 1.00). Consistently, the differential expression analysis revealed that these genes were significantly downregulated in LUSC tumor tissues compared to normal control, suggesting that decreased expression of these proteins plays a role in LUSC development.

### 3.4. Causal Protein Druggability and Associations with Current Medications

**Figure 2:**
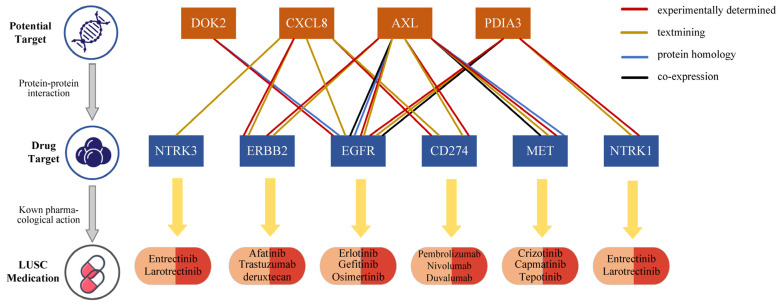
Interaction between current LUSC medications targets and identified potential drug targets.

Interactions between drugs and their molecular targets are essential for drug discovery and therapeutic development. To elucidate the potential mechanisms of action associated with the identified proteins, a PPI network was constructed using the STRING database, integrating these proteins with known LUSC drug targets. The PPI network revealed interactions among four candidate causal proteins (DOK2, CXCL8, AXL, and PDIA3) and six drug targets currently used in LUSC treatment (Figure 2), highlighting the potential of these proteins as promising drug targets for LUSC. Among the identified proteins, DOK2 showed a strong interaction with EGFR, a target for erlotinib, gefitinib, and osimertinib. CXCL8, AXL, and PDIA3 also exhibited strong interactions with multiple drug targets. For instance, AXL revealed strong interactions with EGFR, receptor tyrosine-protein kinase erbB-2 (ERBB2), cluster of differentiation 274/programmed cell death 1 ligand 1 (CD274/PD-L1) and hepatocyte growth factor receptor (MET) (Supplementary Figure S8).

Druggability evaluation of the 12 identified risk proteins revealed that three (AXL, CXCL8, and PDIA3) are already recognized as drug development targets for leukemia, cancer, and inflammation. Notably, the drug gilteritinib, which targets AXL, has received approval for the treatment of acute myeloid leukemia, while AXL1717 is being investigation for NSCLC. Similarly, MDX-018, targeting CXCL8, has shown efficacy in inhibiting tumor growth and treating autoimmune diseases (Supplementary Table S9). These findings underscore the therapeutic relevance of these proteins and their potential to advance LUSC drug development.

### 3.5. Identification of 14 Modifiable Risk Factors as Potential Interventions for LUSC Treatment

**Figure 3:**
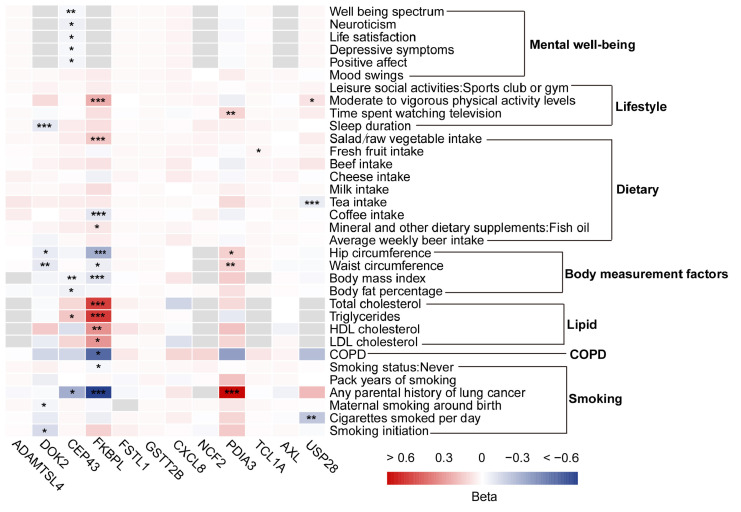
Heatmap of Mendelian randomization between modifiable risk factors and squamous cell lung cancer-related proteins. Significance levels: * False discovery rate corrected *p* < 0.05, ** False discovery rate corrected *p* < 0.01, and *** False discovery rate corrected *p* < 0.001.

To investigate modifiable risk factors associated with LUSC, a two-step MR analysis was performed, evaluating 34 collected risk factors. In the first step, causal effects of the identified plasma proteins on modifiable risk factors were estimated. The identified plasma proteins exhibited significant causal associations with 32 risk factors (Supplementary Table S10), with FKBPL and PDIA3 showed the strongest associations with multiple risk factors (Figure 3). For example, FKBPL was associated with parental history of lung cancer, hip circumference, and vigorous physical activity levels, while PDIA3 was linked to parental history of lung cancer and body mass index (Figure 3 and Supplementary Table S10).

**Figure 4:**
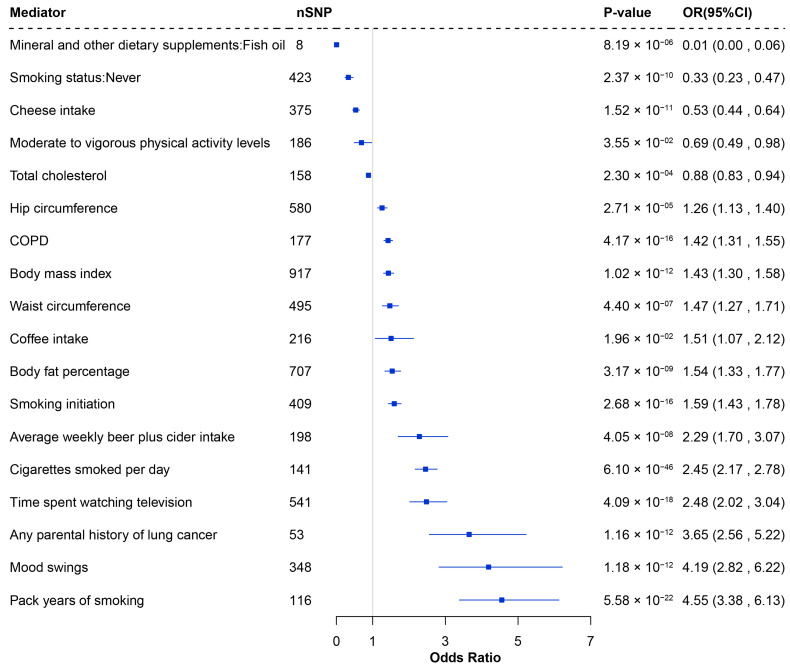
Forest plot of the significant causal effects of modifiable risk factors on LUSC through MR analysis. nSNP: Number of SNP used for the estimation of the causal effects; CI: Confidence interval; OR: Odds ratio; MR: Mendelian randomization.

In the second step, the causal associations between these modifiable risk factors and LUSC were evaluated. A total of 18 risk factors were identified as significantly associated with LUSC. Risk factors such as smoking, average weekly beer intake, COPD, television watching, coffee intake, mood swings, and body measurement factors were associated with an increased risk of LUSC, while fish oil intake, total cholesterol, physical activity, cheese intake, and never smoking reduced risk (Figure 4 and Supplementary Table S11).

By aligning the total effect estimates from the two-step MR analysis with the primary MR analysis, 14 modifiable risk factors were identified as potential mediators, linking six candidate proteins to LUSC (Supplementary Figures S9, S10 and Table S12). Although body measurements had smaller mediation effects, they were consistently implicated in many of the protein-LUSC causal relationships. Notably, parental history of lung cancer was the most significant mediator in the PDIA3-LUSC causal relationship, accounting for 69.46% of the effect and strongly associated with increased LUSC risk (OR = 2.67; 95% CI 1.73–4.11; *p* = 8.83 ×10^−6^). These findings highlight actionable risk factors that could inform targeted interventions for LUSC prevention and treatment.

### 3.6. Computational Assessment of DOK2 Druggability

**Figure 5:**
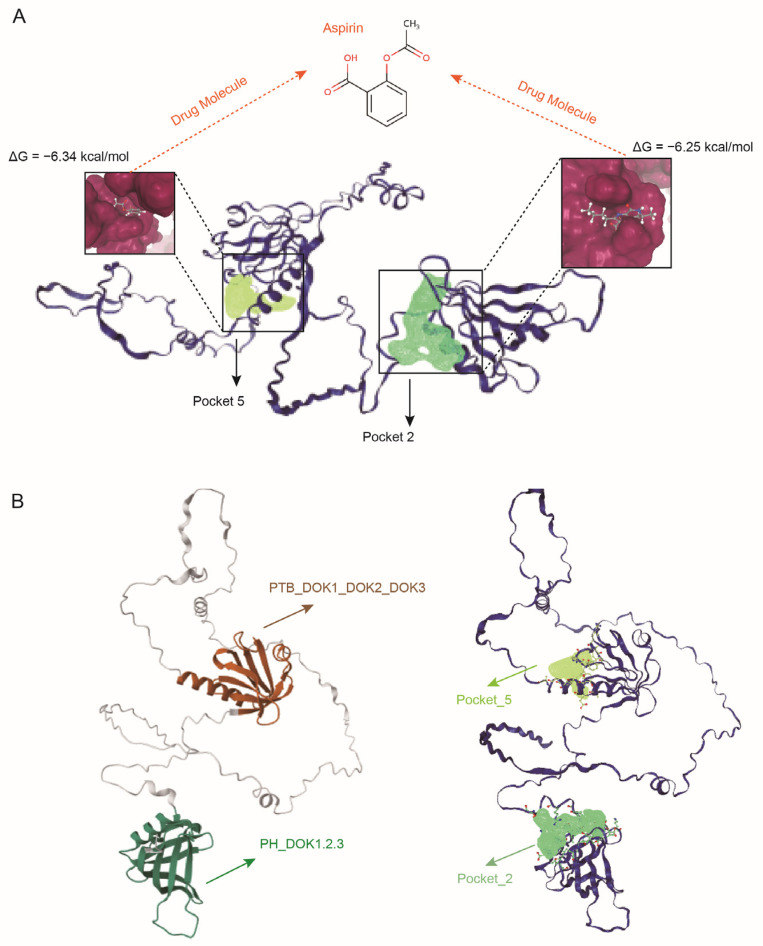
Computational assessment of DOK2 druggability using Aspirin as a probe. (**A**) Molecular docking of Aspirin into the predicted binding pockets. The protein structure is shown in a dark blue cartoon representation. The clinical probe molecule Aspirin is depicted as a gray stick model. The high-scoring drug binding pockets, Pocket 2 and Pocket 5, are highlighted as dark yellow and light green molecular surfaces, respectively. The calculated binding free energy (ΔG) for each stable complex is annotated. (**B**) Localization of the predicted pockets within functional domains. The N-terminal PH domain (residues 7-114, conserved domain: PH_DOK1,2,3) is colored in dark green. The C-terminal PTB domain (residues 147-246, conserved domain: PTB_DOK1_DOK2_DOK3) is colored in dark brown. Structurally, Pocket 2 (light green) is fully embedded within the PH domain, while Pocket 5 (dark yellow) is precisely positioned inside the PTB domain.

To computationally evaluate the druggability of DOK2, we first performed a comprehensive binding pocket scan on its high-confidence AlphaFold2-predicted [40] structure using the DoGSiteScorer algorithm [41]. This scan identified two high-scoring druggable pockets (Pocket 2: drug score 0.82; Pocket 5: drug score 0.81) located within the functionally critical Pleckstrin Homology (PH) and Phosphotyrosine-Binding (PTB) domains (Figure 5A). Furthermore, we validated the binding potential of these pockets through molecular docking studies on the SwissDock [42] platform using Aspirin as a clinical probe molecule. Aspirin was chosen as a probe due to its chemical structure, which comprises a hydrophobic aromatic ring, a hydrogen-bonding carboxyl group, and a flexible acetyl group, thereby demonstrating a promiscuous binding capacity across various protein targets. This characteristic renders it an exemplary molecular ‘master key’ for investigating novel binding sites. Successful docking with Aspirin strongly indicates that the identified pockets exhibit structural versatility, enabling them to accommodate a diverse array of drug-like molecules. The docking results demonstrated stable binding conformations with favorable predicted binding free energies (ΔG = −6.25 kcal/mol for Pocket 2 and −6.34 kcal/mol for Pocket 5), confirming their capacity for high-affinity interactions with drug-like compounds (Figure 5A). Importantly, functional domain analysis (using CDD/SPARCLE [43]) revealed that Pocket 2 within the membrane-targeting PH domain and Pocket 5 within the signal-transducing PTB domain (Figure 5B). This positioning suggests these pockets could serve as allosteric sites for developing agonists that stabilize DOK2 in its active conformation, consistent with the protective role of DOK2 indicated by our MR results. These findings extend beyond simple genetic associations and provide compelling in silico evidence for the ligandability and therapeutic potential of DOK2, offering a more comprehensive basis for its druggability evaluation.

## 4. Discussion

This study leveraged a proteome-wide MR analysis to uncover causal associations between 12 plasma proteins and LUSC risk. Using an integrative approach that include reverse causality detection, colocalization analysis, differential expression analysis, and PPI network analysis, the identified proteins were stratified into four evidence-based target tiers. Specifically, the proteins were categorized as: tier 1 with the strongest evidence (DOK2), tier 2 with strong evidence (FKBPL, NCF2, AXL, and PDIA3), tier 3 with moderate evidence (TCL1A, USP28, CEP43, CXCL8, and ADAMTSL4), and tier 4 with limited evidence (FSTL1 and GSTT2B) (Supplementary Table S13). These classifications provide a framework for prioritizing therapeutic development and advancing LUSC treatment strategies. Furthermore, this study identified the mediating effects of modifiable risk factors on the associations among six plasma proteins and LUSC risk, identifying new avenues for the prevention and treatment of LUSC and underscoring the relevance of targeting both proteins and modifiable risk factors in future therapeutic approaches.

Among the identified proteins, DOK2 emerged as the most promising target, supported by tier 1 classification and strong evidence across multiple analyses. This protein demonstrated significant potential for drug repurposing as a clinical drug candidate for LUSC treatment. The primary MR analysis showed that genetically increased expression of DOK2 was strongly associated with reduced LUSC risk (OR = 0.35; 95% CI 0.23–0.54; *p* = 1.32 ×10^−6^). Specifically, the OR of 0.35 observed for DOK2 in our study indicates a 65% reduction in LUSC risk per standard deviation increase in genetically predicted protein levels. This effect size is substantial in the context of cancer prevention. Furthermore, when a protein demonstrates an OR < 1 in MR analysis, its corresponding drug target would clinically require an agonist to mimic its protective effect. Conversely, developing an antagonist for such a target would be counterproductive and potentially raise safety concerns, making it an unfavorable candidate for drug development. In addition, Differential expression analysis further revealed that *DOK2* gene was significantly down-regulated in LUSC tissues compared with healthy controls, indicating a pivotal role in LUSC. Therefore, the consistency between the direction of effect observed in MR analysis and gene expression data suggests that DOK2 likely functions as an agonist in this context.

DOK2 belongs to the family of tyrosine kinase downstream proteins, which are critical regulators of the receptor tyrosine kinase signaling pathway [44]. Gene *DOK2* is located on chromosome 8p21.3, a genomic region frequently deleted in lung cancer [45]. This gene has been strongly associated with tumor-suppressive functions, and extensive evidence indicated that this chromosomal region represents a reservoir for genes that inhibit tumor growth [46,47,48,49]. Berger et al. identified recurrent loss of copy number in *DOK2* in human lung cancer, correlating with reduced mRNA expression [50]. Additionally, in vivo evidence demonstrated that compound heterozygous deletion of Dok2 and Dusp4 in mice synergistically promotes lung tumorigenesis with short latency and high incidence. Mechanistically, DOK2 and DUSP4 co-deletion was shown to synergistically activate MAPK signaling and drive cell proliferation. Conversely, restoration of these proteins suppressed these oncogenic processes [51,52]. These findings emphasize the critical functional role of DOK2 in inhibiting tumor progression and support its potential as a highlight promising therapeutic target for LUSC.

In addition, our MR findings reveal distinct therapeutic implications for different protein categories. For AXL, despite existing inhibitor development (e.g., bemcentinib), our genetic evidence (OR < 1) suggests caution, as therapeutic inhibition would contradict its protective role. This highlights a critical need to re-evaluate current clinical strategies targeting AXL in LUSC. In contrast, CXCL8 and PDIA3 (both with OR > 1) present promising repurposing opportunities, as their risk-increasing direction aligns perfectly with inhibitor development. For CXCL8, existing antagonists developed for inflammatory conditions could be rapidly evaluated in LUSC contexts. Similarly, PDIA3 inhibitors reported in preclinical studies warrant further investigation as potential LUSC therapeutics. However, significant challenges remain for targets requiring activation. Proteins with OR < 1, including DOK2 and FKBPL, would require agonist development, a process that is considerably more complex than the design of inhibitors. Technical hurdles include achieving cellular penetration for intracellular targets like DOK2 and maintaining pathway specificity to avoid off-target activation. These considerations underscore the importance of aligning therapeutic strategy with genetic evidence while acknowledging the distinct development landscapes for agonists versus inhibitors.

Through comparison with previous proteomic MR studies, we have successfully replicated several established associations. Specifically, TCL1A has been consistently identified as a LUSC-risk protein in two previous pQTL-based MR studies [53,54], and FSTL1 was similarly reported in another recent investigation [55]. This replication across independent studies enhances confidence in these findings. More importantly, our study advances the field through its specific focus on LUSC and utilization of larger, more comprehensive datasets. By employing the latest large-scale LUSC GWAS and the comprehensive UKB-PPP proteomic dataset, we have identified several novel candidates with strong biological support, including DOK2, AXL, NCF2, and FKBPL. Thus, our study demonstrates both consistency with existing findings and provides novel insights through its specific focus on LUSC.

While the current study provides valuable insights, several limitations warrant consideration. First, the study population primarily consisted of individuals of European ancestry to ensure genetic homogeneity, which may limit the generalizability of the findings to other ethnic groups. Second, due to the limited availability of proteomic data from diverse tissues, this analysis focused exclusively on plasma protein levels. Although plasma biomarkers offer significant benefits for non-invasive disease screening and diagnosis, future studies should include proteins from other tissues, particularly tumor tissues, to provide deeper insights into LUSC pathogenesis. Third, despite employing rigorous quality control measures, such as Steiger filtering and sensitivity analyses, residual horizontal pleiotropy and confounding bias cannot be entirely ruled out. Finally, while MR analyses provided robust evidence for causal associations between plasma proteins and LUSC, experimental validation and further studies are essential to substantiate and expand on these findings.

## 5. Conclusions

We identified robust causal relationships between genetically determined plasma proteins and LUSC, with DOK2, FKBPL, NCF2, and AXL emerging as promising therapeutic targets. Furthermore, we revealed the critical role of modifiable risk factors as mediators in the plasma proteins-LUSC relationship, offering opportunities for targeted interventions. These findings provide novel insights into the pathogenic mechanisms underlying LUSC, the development of innovative therapeutic strategies, and the influence of modifiable risk factors in the etiology of LUSC. Future studies should focus on elucidating the functional mechanisms of these candidate proteins and designing intervention strategies that target these modifiable risk factors to effectively reduce LUSC risk.

## Data Availability

All data generated in this study will be available from the corresponding author on reasonable request.

## Supplementary Materials

The following supporting information can be downloaded at: https://www.mdpi.com/article/10.3390/biology14121640/s1, Figure S1: Scatter plots for seven significant MR proteins; Figure S2: Leave-one-out sensitivity analysis plots for four significant MR proteins; Figure S3: Funnel plots for four significant MR proteins; Figure S4: Scatter plots for 18 significant MR risk factors; Figure S5: Leave-one-out sensitivity analysis plots for 18 significant MR risk factors; Figure S6: Funnel plots for 18 significant MR risk factors; Figure S7: Box plot showing differential expression of protein-coding genes identified by MR across different groups; Figure S8: Mediation analysis results of modifiable risk factors in the causal relationship between proteins and LUSC; Figure S9: Mediating effects of modifiable risk factors in the causal association between proteins and LUSC; Figure S10: Venn diagram illustrating the mediating effects of modifiable risk factors in the association between plasma proteins and LUSC; Table S1: Genetic instruments of plasma proteins for MR analysis; Table S2: Summary of modifiable risk factor datasets used in the current study; Table S3: Full significant MR results for LUSC; Table S4: Results of heterogeneity and pleiotropy analysis; Table S5: Results of identified proteins in the bidirectional MR analysis and Steiger filtering; Table S6: Colocalization analysis of LUSC-associated risk proteins; Table S7: Results of differential expression analysis utilizing GEO datasets; Table S8: Results of differential expression analysis utilizing TCGA datasets; Table S9: Druggability assessment of identified LUSC-associated proteins; Table S10: Results of Mendelian randomization for modifiable risk factors and LUSC-related plasma proteins; Table S11: Results of Mendelian randomization for identified modifiable risk factors and LUSC; Table S12: Results of mediation analysis of protein-LUSC via modifiable risk factors; Table S13: Prioritization of Novel Drug Targets for LUSC.

## References

[1] Barta J.A., Powell C.A., Wisnivesky J.P. (2019). Global Epidemiology of Lung Cancer. Ann. Glob. Health.

[2] Siegel R.L., Miller K.D., Fuchs H.E., Jemal A. (2022). Cancer statistics, 2022. CA Cancer J. Clin..

[3] Cooper A.J., Sequist L.V., Lin J.J. (2022). Third-generation EGFR and ALK inhibitors: Mechanisms of resistance and management. Nat. Rev. Clin. Oncol..

[4] Hirsch F.R., Scagliotti G.V., Mulshine J.L., Kwon R., Curran W.J., Wu Y.L., Paz-Ares L. (2017). Lung cancer: Current therapies and new targeted treatments. Lancet.

[5] Majeed U., Manochakian R., Zhao Y., Lou Y. (2021). Targeted therapy in advanced non-small cell lung cancer: Current advances and future trends. J. Hematol. Oncol..

[6] Zhong L., Li Y., Xiong L., Wang W., Wu M., Yuan T., Yang W., Tian C., Miao Z., Wang T. (2021). Small molecules in targeted cancer therapy: Advances, challenges, and future perspectives. Signal Transduct. Target. Ther..

[7] Niu Z., Jin R., Zhang Y., Li H. (2022). Signaling pathways and targeted therapies in lung squamous cell carcinoma: Mechanisms and clinical trials. Signal Transduct. Target. Ther..

[8] Suhre K., Arnold M., Bhagwat A.M., Cotton R.J., Engelke R., Raffler J., Sarwath H., Thareja G., Wahl A., DeLisle R.K. (2017). Connecting genetic risk to disease end points through the human blood plasma proteome. Nat. Commun..

[9] Shi J., Shiraishi K., Choi J., Matsuo K., Chen T.Y., Dai J., Hung R.J., Chen K., Shu X.O., Kim Y.T. (2023). Genome-wide association study of lung adenocarcinoma in East Asia and comparison with a European population. Nat. Commun..

[10] Lindström S., Wang L., Feng H., Majumdar A., Huo S., Macdonald J., Harrison T., Turman C., Chen H., Mancuso N. (2023). Genome-wide analyses characterize shared heritability among cancers and identify novel cancer susceptibility regions. J. Natl. Cancer Inst..

[11] Nelson M.R., Tipney H., Painter J.L., Shen J., Nicoletti P., Shen Y., Floratos A., Sham P.C., Li M.J., Wang J. (2015). The support of human genetic evidence for approved drug indications. Nat. Genet..

[12] Reay W.R., Cairns M.J. (2021). Advancing the use of genome-wide association studies for drug repurposing. Nat. Rev. Genet..

[13] Richmond R.C., Davey Smith G. (2022). Mendelian Randomization: Concepts and Scope. Cold Spring Harb. Perspect. Med..

[14] McKay J.D., Hung R.J., Han Y., Zong X., Carreras-Torres R., Christiani D.C., Caporaso N.E., Johansson M., Xiao X., Li Y. (2017). Large-scale association analysis identifies new lung cancer susceptibility loci and heterogeneity in genetic susceptibility across histological subtypes. Nat. Genet..

[15] Timofeeva M.N., Hung R.J., Rafnar T., Christiani D.C., Field J.K., Bickeböller H., Risch A., McKay J.D., Wang Y., Dai J. (2012). Influence of common genetic variation on lung cancer risk: Meta-analysis of 14,900 cases and 29,485 controls. Hum. Mol. Genet..

[16] Wang Y., McKay J.D., Rafnar T., Wang Z., Timofeeva M.N., Broderick P., Zong X., Laplana M., Wei Y., Han Y. (2014). Rare variants of large effect in BRCA2 and CHEK2 affect risk of lung cancer. Nat. Genet..

[17] Wang Y., Wei Y., Gaborieau V., Shi J., Han Y., Timofeeva M.N., Su L., Li Y., Eisen T., Amos C.I. (2015). Deciphering associations for lung cancer risk through imputation and analysis of 12,316 cases and 16,831 controls. Eur. J. Hum. Genet. EJHG.

[18] Sun B.B., Chiou J., Traylor M., Benner C., Hsu Y.H., Richardson T.G., Surendran P., Mahajan A., Robins C., Vasquez-Grinnell S.G. (2023). Plasma proteomic associations with genetics and health in the UK Biobank. Nature.

[19] Malhotra J., Malvezzi M., Negri E., La Vecchia C., Boffetta P. (2016). Risk factors for lung cancer worldwide. Eur. Respir. J..

[20] Emdin C.A., Khera A.V., Kathiresan S. (2017). Mendelian Randomization. Jama.

[21] Davey Smith G., Hemani G. (2014). Mendelian randomization: Genetic anchors for causal inference in epidemiological studies. Hum. Mol. Genet..

[22] Bowden J., Davey Smith G., Haycock P.C., Burgess S. (2016). Consistent Estimation in Mendelian Randomization with Some Invalid Instruments Using a Weighted Median Estimator. Genet. Epidemiol..

[23] Schmidt A.F., Dudbridge F. (2018). Mendelian randomization with Egger pleiotropy correction and weakly informative Bayesian priors. Int. J. Epidemiol..

[24] Hemani G., Tilling K., Davey Smith G. (2017). Orienting the causal relationship between imprecisely measured traits using GWAS summary data. PLoS Genet..

[25] Giambartolomei C., Vukcevic D., Schadt E.E., Franke L., Hingorani A.D., Wallace C., Plagnol V. (2014). Bayesian test for colocalisation between pairs of genetic association studies using summary statistics. PLoS Genet..

[26] Liu B., Gloudemans M.J., Rao A.S., Ingelsson E., Montgomery S.B. (2019). Abundant associations with gene expression complicate GWAS follow-up. Nat. Genet..

[27] Imyanitov E.N., Iyevleva A.G., Levchenko E.V. (2021). Molecular testing and targeted therapy for non-small cell lung cancer: Current status and perspectives. Crit. Rev. Oncol. Hematol..

[28] Araghi M., Mannani R., Heidarnejad Maleki A., Hamidi A., Rostami S., Safa S.H., Faramarzi F., Khorasani S., Alimohammadi M., Tahmasebi S. (2023). Recent advances in non-small cell lung cancer targeted therapy; an update review. Cancer Cell Int..

[29] Lee J.M., McNamee C.J., Toloza E., Negrao M.V., Lin J., Shum E., Cummings A.L., Kris M.G., Sepesi B., Bara I. (2023). Neoadjuvant Targeted Therapy in Resectable NSCLC: Current and Future Perspectives. J. Thorac. Oncol. Off. Publ. Int. Assoc. Study Lung Cancer.

[30] Mendez D., Gaulton A., Bento A.P., Chambers J., De Veij M., Félix E., Magariños M.P., Mosquera J.F., Mutowo P., Nowotka M. (2019). ChEMBL: Towards direct deposition of bioassay data. Nucleic Acids Res..

[31] Freshour S.L., Kiwala S., Cotto K.C., Coffman A.C., McMichael J.F., Song J.J., Griffith M., Griffith O.L., Wagner A.H. (2021). Integration of the Drug-Gene Interaction Database (DGIdb 4.0) with open crowdsource efforts. Nucleic Acids Res..

[32] Wishart D.S., Feunang Y.D., Guo A.C., Lo E.J., Marcu A., Grant J.R., Sajed T., Johnson D., Li C., Sayeeda Z. (2018). DrugBank 5.0: A major update to the DrugBank database for 2018. Nucleic Acids Res..

[33] Rousseaux S., Debernardi A., Jacquiau B., Vitte A.L., Vesin A., Nagy-Mignotte H., Moro-Sibilot D., Brichon P.Y., Lantuejoul S., Hainaut P. (2013). Ectopic activation of germline and placental genes identifies aggressive metastasis-prone lung cancers. Sci. Transl. Med..

[34] Ritchie M.E., Phipson B., Wu D., Hu Y., Law C.W., Shi W., Smyth G.K. (2015). limma powers differential expression analyses for RNA-sequencing and microarray studies. Nucleic Acids Res..

[35] de Groot P.M., Wu C.C., Carter B.W., Munden R.F. (2018). The epidemiology of lung cancer. Transl. Lung Cancer Res..

[36] Relton C.L., Davey Smith G. (2012). Two-step epigenetic Mendelian randomization: A strategy for establishing the causal role of epigenetic processes in pathways to disease. Int. J. Epidemiol..

[37] VanderWeele T.J. (2016). Mediation Analysis: A Practitioner’s Guide. Annu. Rev. Public Health.

[38] Carter A.R., Sanderson E., Hammerton G., Richmond R.C., Davey Smith G., Heron J., Taylor A.E., Davies N.M., Howe L.D. (2021). Mendelian randomisation for mediation analysis: Current methods and challenges for implementation. Eur. J. Epidemiol..

[39] Zheng J., Haberland V., Baird D., Walker V., Haycock P.C., Hurle M.R., Gutteridge A., Erola P., Liu Y., Luo S. (2020). Phenome-wide Mendelian randomization mapping the influence of the plasma proteome on complex diseases. Nat. Genet..

[40] Jumper J., Evans R., Pritzel A., Green T., Figurnov M., Ronneberger O., Tunyasuvunakool K., Bates R., Žídek A., Potapenko A. (2021). Highly accurate protein structure prediction with AlphaFold. Nature.

[41] Graef J., Ehrt C., Rarey M. (2023). Binding Site Detection Remastered: Enabling Fast, Robust, and Reliable Binding Site Detection and Descriptor Calculation with DoGSite3. J. Chem. Inf. Model..

[42] Bugnon M., Röhrig U.F., Goullieux M., Perez M.A.S., Daina A., Michielin O., Zoete V. (2024). SwissDock 2024: Major enhancements for small-molecule docking with Attracting Cavities and AutoDock Vina. Nucleic Acids Res..

[43] Lu S., Wang J., Chitsaz F., Derbyshire M.K., Geer R.C., Gonzales N.R., Gwadz M., Hurwitz D.I., Marchler G.H., Song J.S. (2020). CDD/SPARCLE: The conserved domain database in 2020. Nucleic Acids Res..

[44] Janas J.A., Van Aelst L. (2011). Oncogenic tyrosine kinases target Dok-1 for ubiquitin-mediated proteasomal degradation to promote cell transformation. Mol. Cell. Biol..

[45] Chang J., Tian J., Zhu Y., Zhong R., Zhai K., Li J., Ke J., Han Q., Lou J., Chen W. (2018). Exome-wide analysis identifies three low-frequency missense variants associated with pancreatic cancer risk in Chinese populations. Nat. Commun..

[46] Chitale D., Gong Y., Taylor B.S., Broderick S., Brennan C., Somwar R., Golas B., Wang L., Motoi N., Szoke J. (2009). An integrated genomic analysis of lung cancer reveals loss of DUSP4 in EGFR-mutant tumors. Oncogene.

[47] Weir B.A., Woo M.S., Getz G., Perner S., Ding L., Beroukhim R., Lin W.M., Province M.A., Kraja A., Johnson L.A. (2007). Characterizing the cancer genome in lung adenocarcinoma. Nature.

[48] Wistuba, Behrens C., Virmani A.K., Milchgrub S., Syed S., Lam S., Mackay B., Minna J.D., Gazdar A.F. (1999). Allelic losses at chromosome 8p21-23 are early and frequent events in the pathogenesis of lung cancer. Cancer Res..

[49] Yamanashi Y., Baltimore D. (1997). Identification of the Abl- and rasGAP-associated 62 kDa protein as a docking protein, Dok. Cell.

[50] Berger A.H., Niki M., Morotti A., Taylor B.S., Socci N.D., Viale A., Brennan C., Szoke J., Motoi N., Rothman P.B. (2010). Identification of DOK genes as lung tumor suppressors. Nat. Genet..

[51] Guan Y., Li M., Qiu Z., Xu J., Zhang Y., Hu N., Zhang X., Guo W., Yuan J., Shi Q. (2022). Comprehensive analysis of DOK family genes expression, immune characteristics, and drug sensitivity in human tumors. J. Adv. Res..

[52] Chen M., Zhang J., Berger A.H., Diolombi M.S., Ng C., Fung J., Bronson R.T., Castillo-Martin M., Thin T.H., Cordon-Cardo C. (2019). Compound haploinsufficiency of Dok2 and Dusp4 promotes lung tumorigenesis. J. Clin. Investig..

[53] Chen Y., Dai X., Zhang P., Yao L., Wang Q., Qin Z., Gao T., Zhang J. (2025). Proteome-wide Mendelian randomization and colocalization analysis uncovers druggable targets for lung cancer across multiple phenotypes and complications. Discov. Oncol..

[54] Dong B., Wang M., Li K., Li Z., Liu L., Shen S. (2024). Plasma proteometabolome in lung cancer: Exploring biomarkers through bidirectional Mendelian randomization and colocalization analysis. Hum. Mol. Genet..

[55] Feng Y., Li C., Cheng B., Chen Y., Chen P., Wang Z., Zheng X., He J., Zhu F., Wang W. (2024). Identifying genetically-supported drug repurposing targets for non-small cell lung cancer through mendelian randomization of the druggable genome. Transl. Lung Cancer Res..

