# Supplementary material for: Integrating Human Proteomes with Genome-Wide Association Data Reveals Prioritized Therapeutic Candidates for Lung Squamous Cell Carcinoma
Source: Biology (Basel). 2025 Nov 21;14(12):1640. doi: 10.3390/biology14121640 (PMC12730066; doi:10.3390/biology14121640)
Supplement: Supplementary file 1 [file biology-14-01640-s001.zip › Supplementary Material-Analytical Workflow-Updated.pdf]

Detailed Step-by-Step Description

Step 1: Data Preparation and Harmonization

- **1.1. Exposure Data (pQTLs):**
  - **Source:** UK Biobank Pharma Proteomics Project (UKB-PPP).
  - **Filtering Criteria:**
    - Significance: $P < 5e-8$
    - Region: Excluded MHC region (chr6:26-34Mb, hg19).
    - Independence: LD clumping ($r^2 < 0.001$).
    - Type: Retained only *cis*-acting pQTLs.
  - **Outcome:** 6,202 independent SNPs for 1,972 proteins.
  - **Tool:** Standard GWAS QC pipelines and PLINK for clumping.
- **1.2. Outcome Data (LUSC):**
  - **Source:** GWAS summary statistics from TRICL consortium (McKay et al., 2017).
  - **Sample:** 7,426 cases and 55,627 controls.
  - **Harmonization:** Aligned all SNPs to the forward strand. Palindromic SNPs were excluded if allele frequency was ambiguous. Ensured the effect allele for exposure and outcome datasets referred to the same allele.
- **1.3. Software & Package:**
  - R packages: TwoSampleMR (v0.6.1) was used for data harmonization.

Step 2: Primary Mendelian Randomization Analysis

- **2.1. Method Selection:**
  - For proteins with **one IV**: Wald ratio method.
  - For proteins with **multiple IVs**: Inverse variance weighted (IVW) method as the primary analysis.
- **2.2. Multiple Testing Correction:**
  - Bonferroni correction: Significance threshold set at $P < 0.05 / 1972 \approx 2.54e-5$.
- **2.3. Software & Package:**
  - R package: TwoSampleMR (v0.6.1). The core function used was mr() with the specified method.

Step 3: Sensitivity and Robustness Analyses

- **3.1. Heterogeneity Test:**
  - **Method:** Cochran's Q statistic (from IVW method).
  - **Interpretation:** $P_Q > 0.05$ indicates no significant heterogeneity.
- **3.2. Horizontal Pleiotropy Test:**
  - **Method:** MR-Egger regression intercept test.
  - **Interpretation:** $P_{intercept} > 0.05$ indicates no significant directional pleiotropy.
- **3.3. Reverse Causality Detection:**
  - **Method 1 (Bidirectional MR):** Swapped exposure and outcome. Significance threshold: $P < 0.05$.
  - **Method 2 (Steiger Filtering):** Used directionality_test() in TwoSampleMR to ensure the SNP-exposure association was stronger than the SNP-outcome association.
- **3.4. Software & Package:**
  - R package: TwoSampleMR (v0.6.1).

Functions: mr_heterogeneity(), mr_pleiotropy_test(), directionality_test().

Step 4: Bayesian Colocalization Analysis

- **4.1. Objective:** To assess if the pQTL and LUSC GWAS signals share a common causal variant.
- **4.2. Locus Definition:** cis-region of the protein-coding gene ± 500 kb.
- **4.3. Method & Priors:** Used default priors in the coloc package (p1 = 1e-4, p2 = 1e-4, p12 = 1e-5).
- **4.4. Threshold for Evidence:**
  - Strong evidence: Posterior Probability for H4 (PP.H4) ≥ 0.75.
  - Moderate evidence: PP.H4 ≥ 0.60.
- **4.5. Software & Package:**
  - R package: coloc (v5.3.2). The core function used was coloc.abf().

Step 5. Target Prioritization and Validation

- **5.1. Differential Expression Analysis:**
  - **Data Sources:** TCGA-LUSC and GEO (GSE30219).
  - **Tool:** limma package (v3.58.1) in R.
  - **Threshold:** |Log2FC| > 1 and adjusted P < 0.05.
- **5.2. Protein-Protein Interaction (PPI) Network:**
  - **Tool:** STRING database (v12.0).
  - **Parameters:** Minimum required interaction score: 0.40.
- **5.3. Druggability Assessment:**
  - **Databases Queried:** DrugBank, ChEMBL, DGIdb.

Step 6. Mediation Analysis

- **6.1. Objective:** To identify if modifiable risk factors mediate the protein-LUSC relationship.
- **6.2. Method:** Two-step MR.
  - **Step 1:** MR (Protein → Risk Factor).
  - **Step 2:** MR (Risk Factor → LUSC).
- **6.3. Mediation Effect Calculation:**
  - **Method:** Product of coefficients method.
  - **Variance Estimation:** Delta method.
- **6.4. Software & Package:**
  - R package: TwoSampleMR for MR steps. Custom R script for mediation calculation.

Note on Code Availability: *The specific analysis code is available upon reasonable request from the corresponding author, subject to the data use agreements of the original GWAS and proteomics consortia.*
