# Supplementary material for: Integrating Human Proteomes with Genome-Wide Association Data Reveals Prioritized Therapeutic Candidates for Lung Squamous Cell Carcinoma
Source: Biology (Basel). 2025 Nov 21;14(12):1640. doi: 10.3390/biology14121640 (PMC12730066; doi:10.3390/biology14121640)
Supplement: Supplementary file 1 [file biology-14-01640-s001.zip › SupplementaryMaterials.pdf]

Supplementary Figures

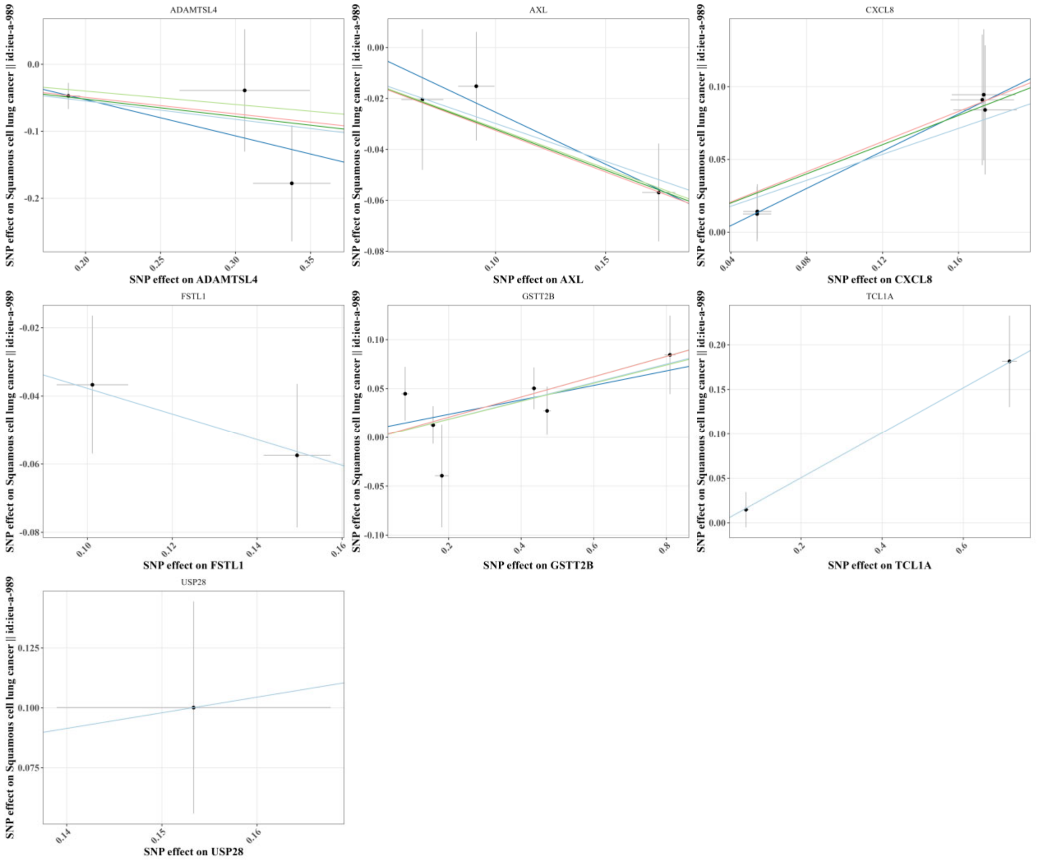

Supplementary Figure 1. Scatter plots for seven significant MR proteins. Each point represents a genetic variant (SNP) used as an instrumental variable. Slope lines indicate causal effect estimates from different Mendelian randomization methods. Light blue line: Inverse-variance weighted (IVW); Green line: Weighted median; Dark blue line: MR-Egger regression; Red line: Weighted mode; Light green line: Simple mode; Error bars represent 95% confidence intervals.

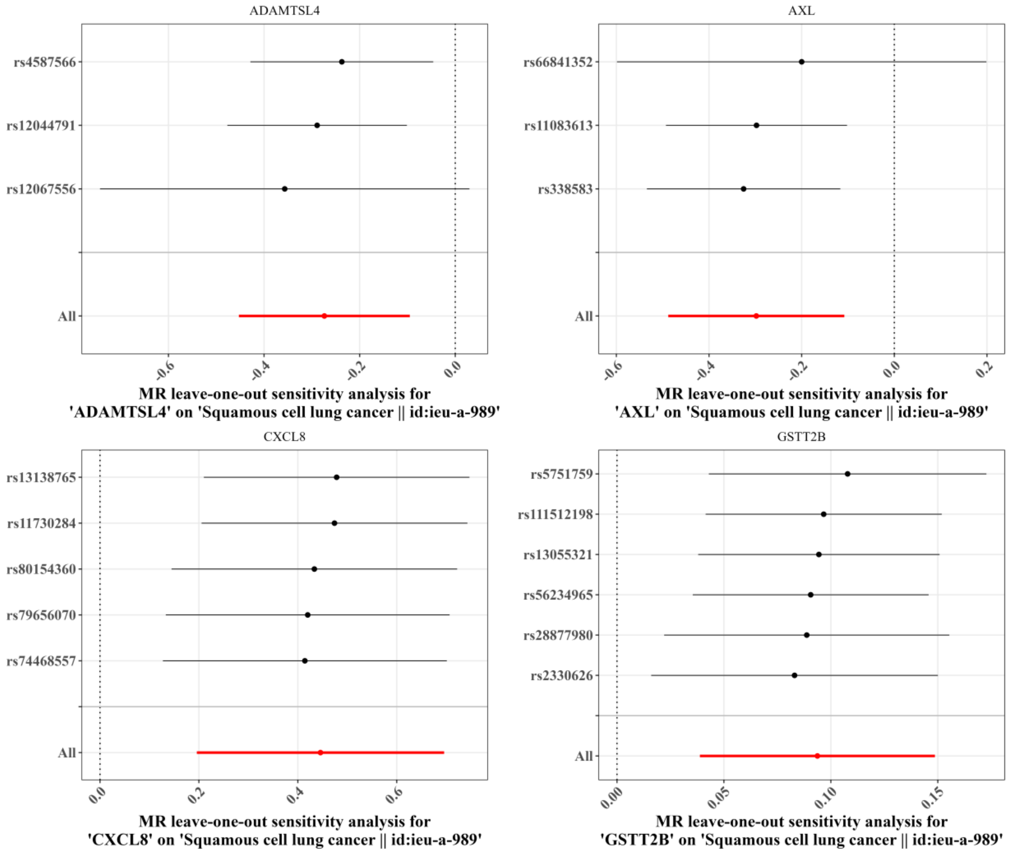

Supplementary Figure 2. Leave-one-out sensitivity analysis plots for four significant MR proteins. Each point shows the IVW estimate after excluding the corresponding SNP, with error bars representing 95% confidence intervals.

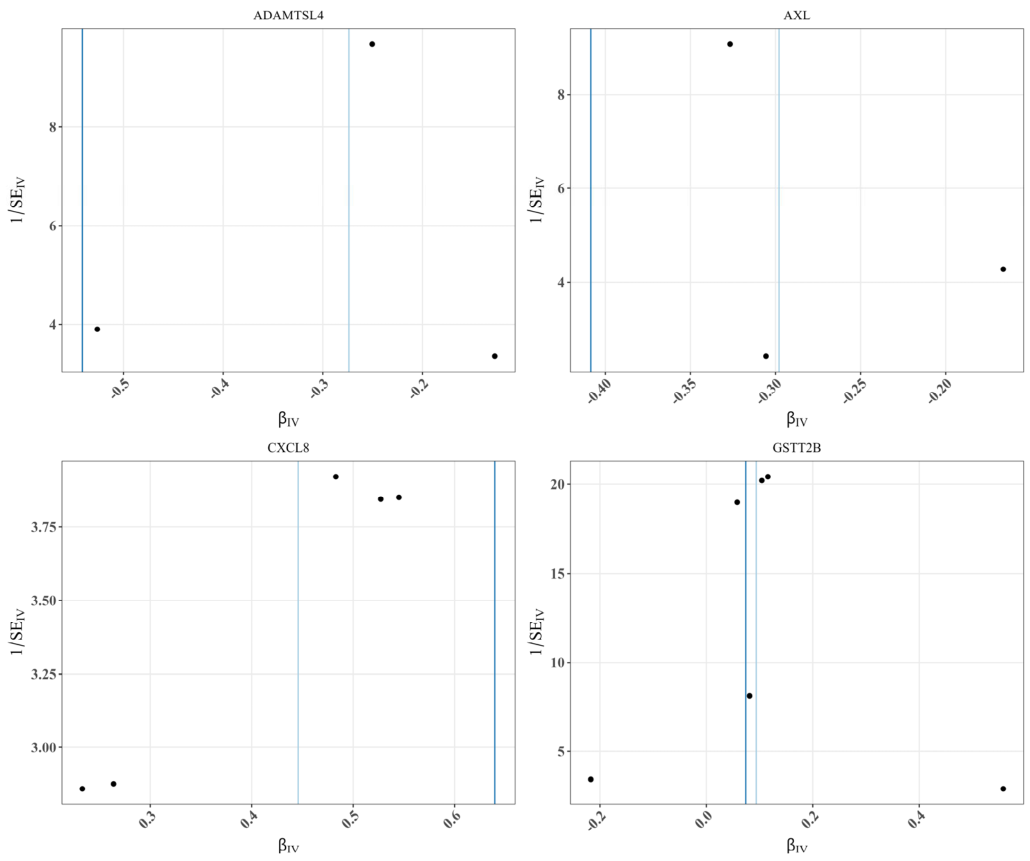

Supplementary Figure 3. Funnel plots for four significant MR proteins. Each point represents causal estimates from individual variants plotted against precision. Light blue dashed line: Null effect line; Dark blue solid line: IVW pooled estimate.

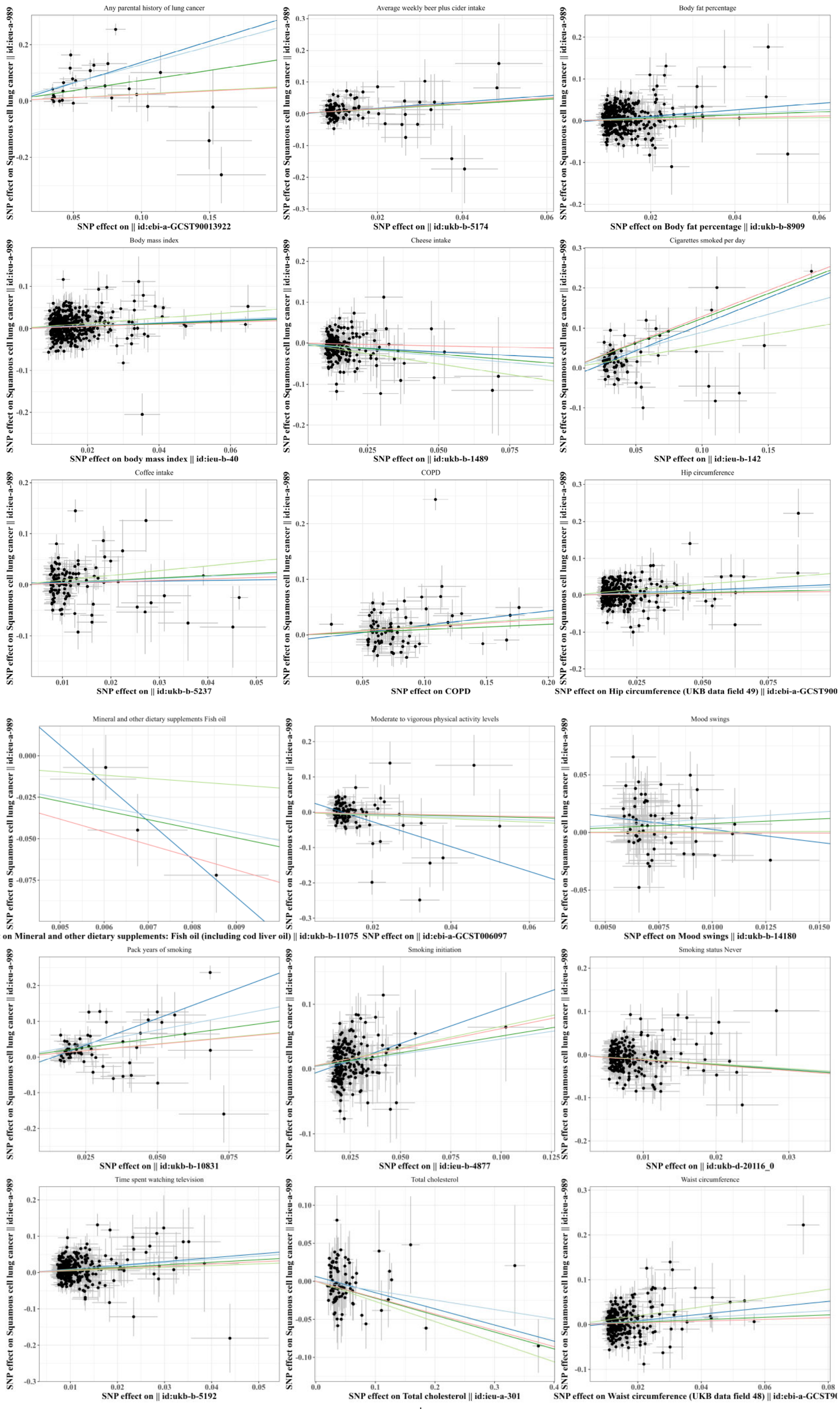

Any parental history of lung cancer
SNP effect on Squamous cell lung cancer || id:ieu-a-989
SNP effect on || id:ebi-a-GCST90013922
Average weekly beer plus cider intake
SNP effect on || id:ukb-b-5174
Body fat percentage
SNP effect on Body fat percentage || id:ukb-b-8909
Body mass index
SNP effect on body mass index || id:ieu-b-40
Cheese intake
SNP effect on || id:ukb-b-1489
Cigarettes smoked per day
SNP effect on || id:ieu-b-142
Coffee intake
SNP effect on || id:ukb-b-5237
COPD
SNP effect on COPD
Hip circumference
SNP effect on Hip circumference (UKB data field 49) || id:ebi-a-GCST900
Mineral and other dietary supplements Fish oil
on Mineral and other dietary supplements: Fish oil (including cod liver oil) || id:ukb-b-11075
Moderate to vigorous physical activity levels
SNP effect on || id:ebi-a-GCST006097
Mood swings
SNP effect on Mood swings || id:ukb-b-14180
Pack years of smoking
SNP effect on || id:ukb-b-10831
Smoking initiation
SNP effect on || id:ieu-b-4877
Smoking status Never
SNP effect on || id:ukb-d-20116_0
Time spent watching television
SNP effect on || id:ukb-b-5192
Total cholesterol
SNP effect on Total cholesterol || id:ieu-a-301
Waist circumference
SNP effect on Waist circumference (UKB data field 48) || id:ebi-a-GCST9(

Supplementary Figure 4. Scatter plots for 18 significant MR risk factors. Each point represents a genetic variant (SNP) used as an instrumental variable. Slope lines indicate causal effect estimates from different Mendelian randomization methods. Light blue line: Inverse-variance weighted (IVW); Green line: Weighted median; Dark blue line: MR-Egger regression; Red line: Weighted mode; Light green line: Simple mode; Error bars represent 95% confidence intervals.

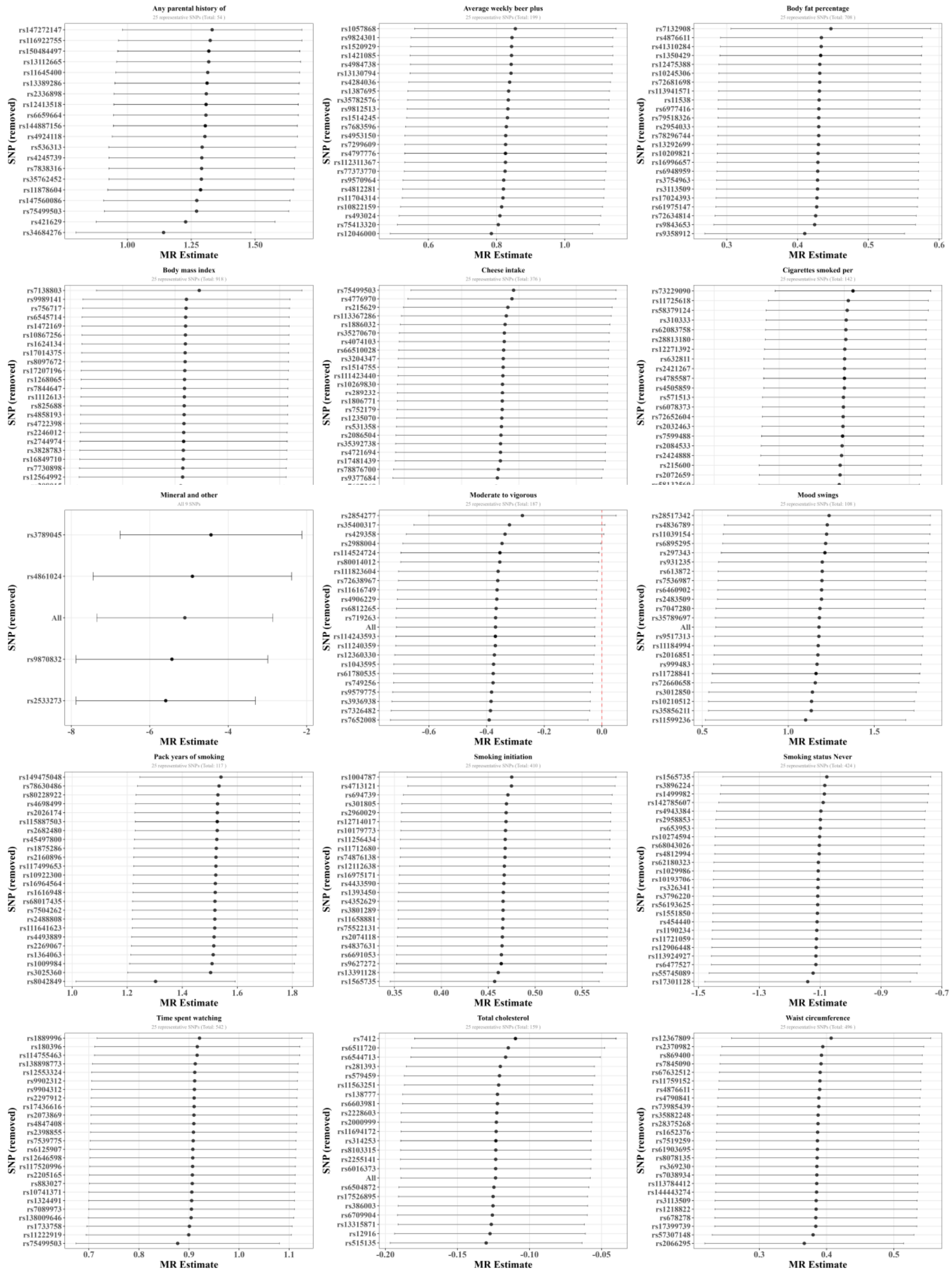

Supplementary Figure 5. Leave-one-out sensitivity analysis plots for 18 significant MR risk factors. Each point shows the IVW estimate after excluding the corresponding SNP, with error bars representing 95% confidence intervals.

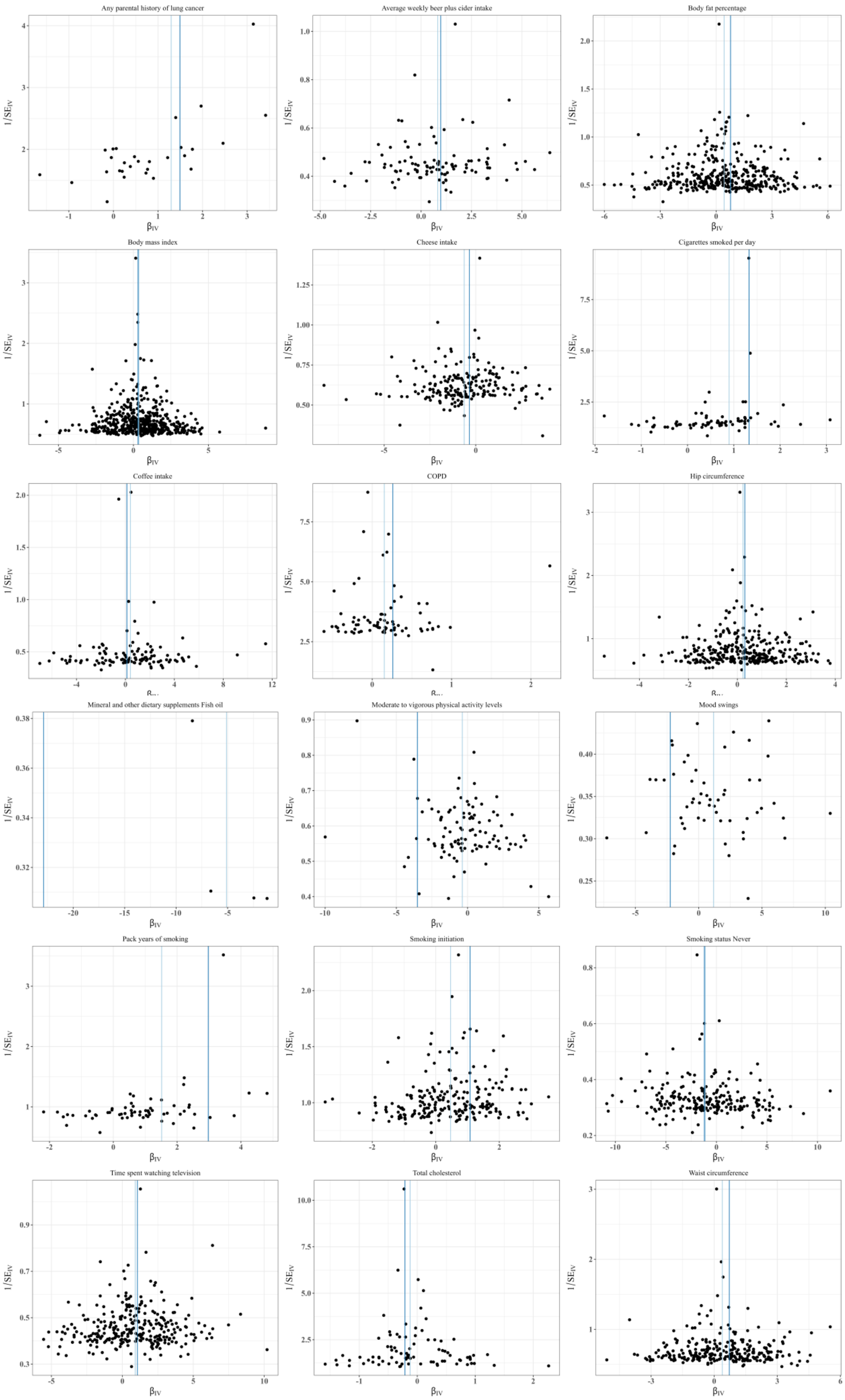
Any parental history of lung cancer
Average weekly beer plus cider intake
Body fat percentage
Body mass index
Cheese intake
Cigarettes smoked per day
Coffee intake
COPD
Hip circumference
Mineral and other dietary supplements Fish oil
Moderate to vigorous physical activity levels
Mood swings
Pack years of smoking
Smoking initiation
Smoking status Never
Time spent watching television
Total cholesterol
Waist circumference
1/SE_IV
β_IV

Supplementary Figure 6. Funnel plots for 18 significant MR risk factors. Each point represents causal estimates from individual variants plotted against precision. Light blue dashed line: Null effect line; Dark blue solid line: IVW pooled estimate.

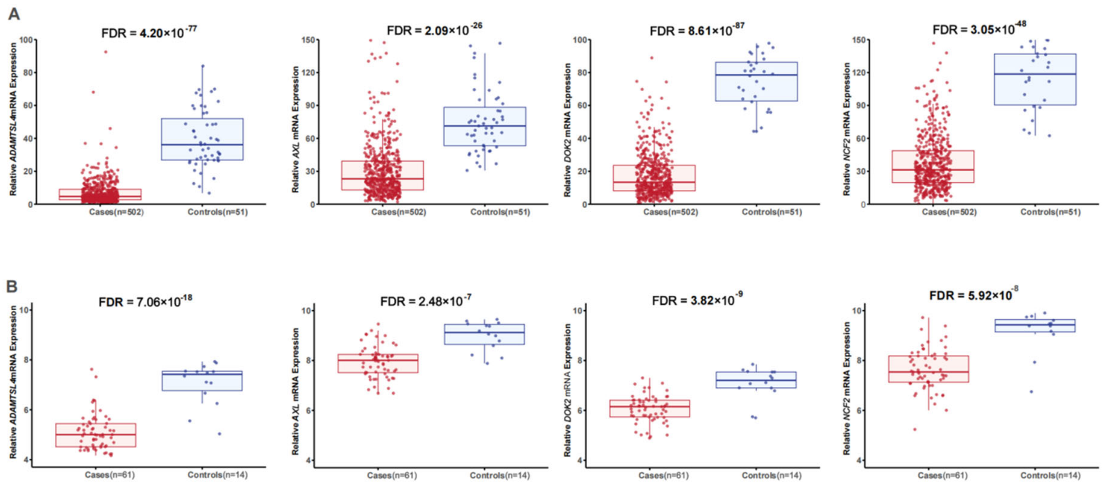

Supplementary Figure 7. Box plot showing differential expression of protein-coding genes s identified by MR across different groups. (A) Differential expression analysis utilizing TCGA datasets. (B) Differential expression analysis utilizing GEO datasets.

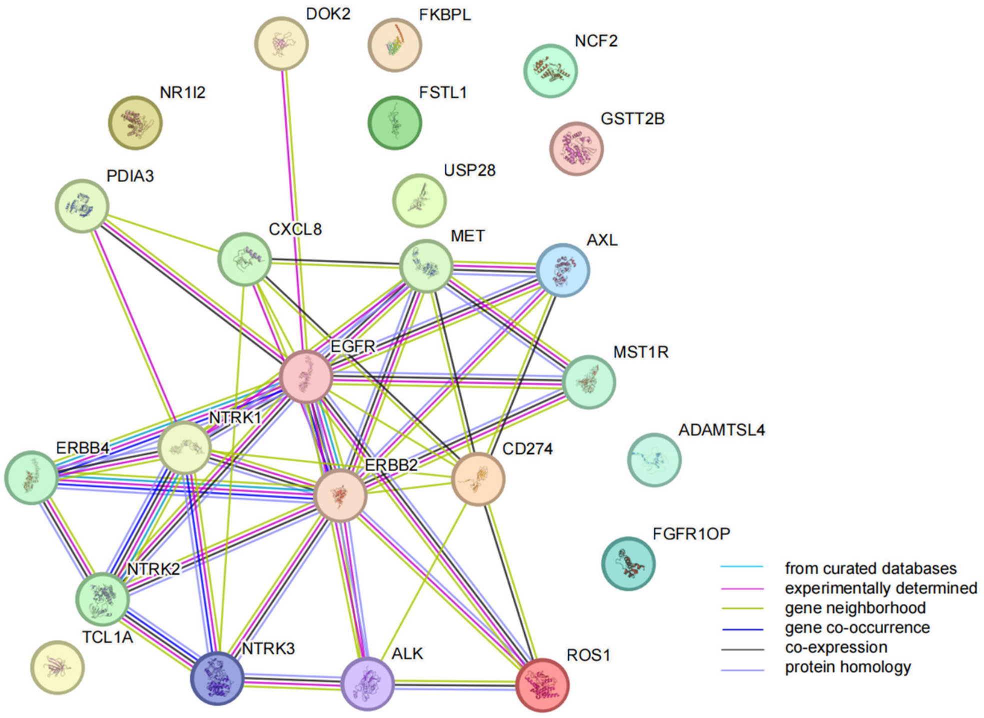

Supplementary Figure 8. Mediation analysis results of modifiable risk factors in the causal relationship between proteins and LUSC.

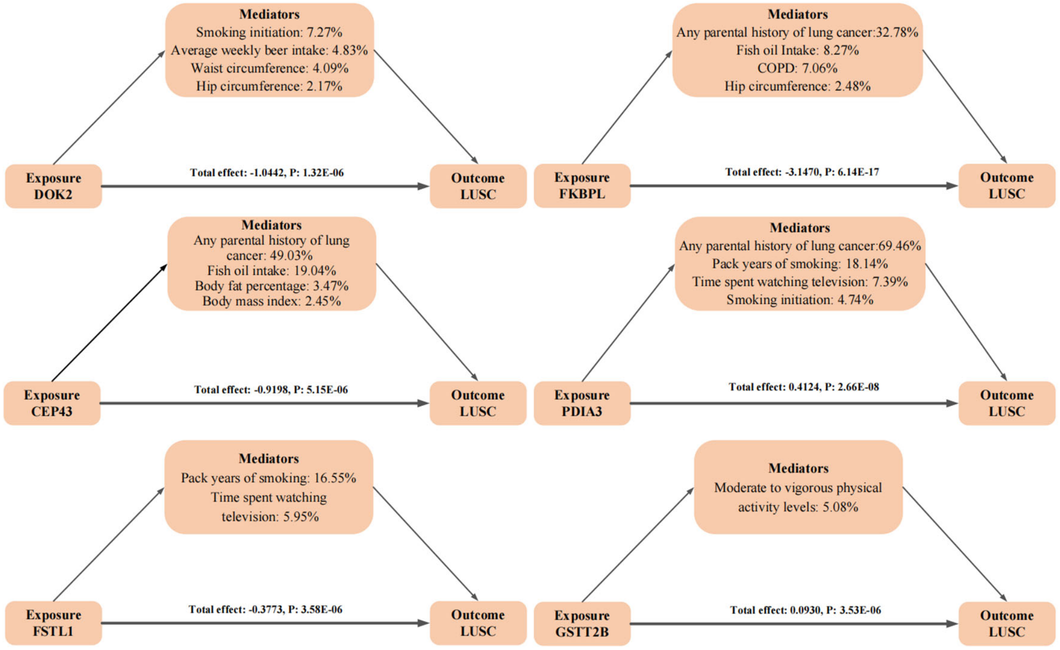

Supplementary Figure 9. Figure showing the mediating effects of modifiable risk factors in the causal association between proteins and LUSC.

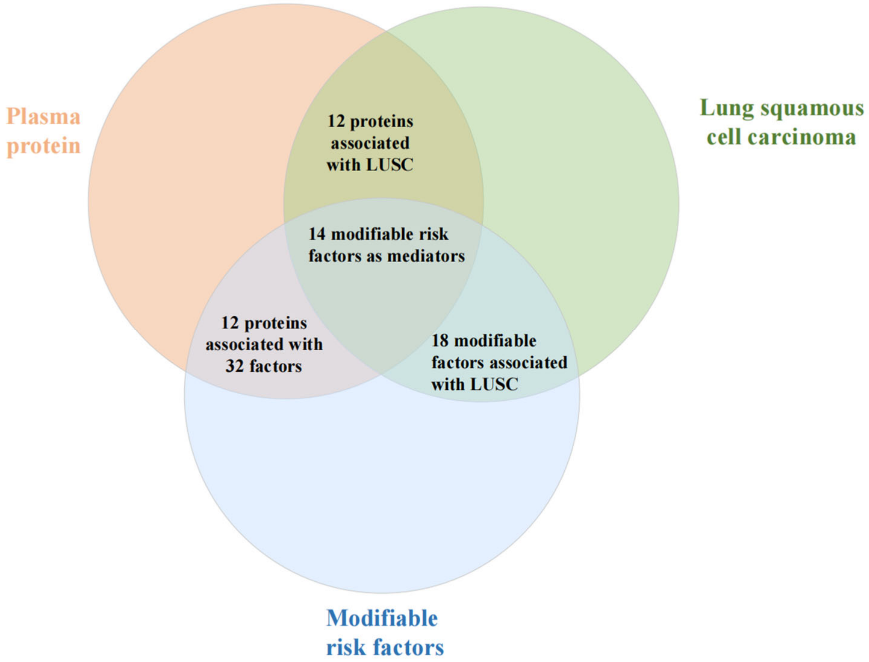

Supplementary Figure 10. Venn diagram illustrating the mediating effects of modifiable risk factors in the association between plasma proteins and LUSC.
